# Miscellaneous

**Published:** 1891-06

**Authors:** S. H. Preston


### MISCELLANEOUS.

### HE WAS NOT MARSHAL NEY.

BY S. H. PRESTON.

From 1816 to 1846, a man known as Peter Ney, lived in Rowan County, North Carolina. He was a cultured, non-communicative man, and some of his neighbors surmised that his life was one of mystery. He followed the profession of teaching school. Sometimes he would get the worse for wine. Though remark ably reticent when sober, he was quite loquacious when in liquor. At those times he would confidentially tell his friends that his real name was Michael Ney —that in fact he was the famous French Marshal, the hero of five hundred battles, who was called "Bravest of the Brave," by Bonaparte, and made Prince of Moskwa by him on the bloody field of Borodino, who covered the retreat of the Grand Army from Moscow and commanded the last charge of the Old Guard at Waterloo.

But after the downfall of Napoleon, Marshal Ney was condemned to death and shot in the garden of the Luxembourg, where a monument marks the spot of his execution. Not so, said the North Carolina Ney. He escaped. The soldiers sent to shoot him chanced to belong to his old command. He only had to tell them to aim high and to drop when the volley was fired. He was duly declared dead and his body was taken away by friends, who secretly sent him to Bordeaux, where he took ship to Charleston, S. C.

And his North Carolina neighbors began to believe this talk of the intemperate teacher. They thought he looked like pictures they had seen of the Marshal. And for sixty years there have been people in the Carolinas who have believed that this Peter Ney was Michael Ney, the hero of a hundred battle fields of Bonaparte. And now the Rev. James A. Weston, a scholarly Episcopal clergyman of Hickory, N. C., has published a pretentious book to prove the claims of Peter.

It is time this silly story was shelved with those respecting the survival of Restell, Wilkes Booth and Brigham Young. Still there will always be folks ready to swallow any improbable tale that is tinged with romantic mystery. Tell them Bonaparte was not sent to St. Helena, but escaped and became a bartender in Boston, or that John Brown is still roaming about the Adirondacks, and they would believe the stories and strive to make them plausible.

A few facts suffice to do away with this Ney myth, that is, among the well informed. Members of his family have never taken any stock in it. On the contrary, they have repeatedly refuted it. They, as well as Napoleon and his old companions in arms, would certainly have known if by any chance Marshal Ney had escaped execution.

And nothing could be more absurd than to suppose that he did escape. The allies, that is, the combined kings of Europe, had determined that he must die. He who had so often routed their armies and came so near overthrowing their thrones at Waterloo, was fixed upon as the victim of their vindictive revenge. The Chamber of Peers condemned him to death by a majority of one hundred and fifty-two.

He was aroused from a placid repose to receive his sentence. As the officer proceeded to recite his numerous titles he cut him short with, "Simply say Michael Ney—once a French soldier and soon a heap of dust." While his wife was on her knees before the king praying for his pardon he was taken out to be shot. To the officer sent to bandage his eyes he said, "Are you ignorant that for twenty-five years I have been accustomed to face both ball and bullets?" He calmly removed his hat, and surveying the squad of soldiers drawn up to shoot him with the same serenity he had always shown in the storm of battle, he raised his right hand to his heart and said, "Comrades, fire on me." He dropped dead, pierced by ten bullets. "Thus," remarks Napier, "he who had fought five hundred battles for France—not one against her—was shot as a traitor."

Bourrienne, Montholon, and others, acquainted with all the details of his death, have furnished full descriptions of it Napoleon grieved greatly over the execution of his faithful friend and favorite Marshal, and at St. Helena told all about it. It is absurd to suppose that his royal enemies who had taken such pains to have him condemned to death would not have guarded against any possibility of escape.

But even had he been saved, as stated in the North Carolina story, there was no occasion for concealing his identity after reaching the United States. He would have been as safe on our shores as Talleyrand was after his flight from England. And there was no need for him to remain here after amnesty had been granted by Louis XVIII., Charles X., and Louis Phillipe. He could have returned to France in absolute security, and he would have been welcomed with an outburst of enthusiasm like that evoked by the arrival of Napoleon's remains from St. Helena.

But even more marvelous than his escape from execution would have been his ability to assume the duties of school instructor upon his arrival in America, being not only unschooled himself, but unacquainted with our language. A professor might pass himself off as an ignorant soldier, but an unlettered man could never play the part of teacher, especially in a foreign land.

Marshal Ney was the son of a country cooper, and commenced his military career at the age of seventeen. He was an uneducated man, and so unaccustomed to the usages of cultured society that in intervals of peace he avoided his elegant palace at Paris, where his wife, given to gaiety and luxury, entertained her aristocratic friends. While Napoleon was in exile at Elba, Ney retired to his rural residence, where he relieved his restlessness in hunting and sports of the field.

Marshal Ney was notably the ablest and grandest of that astonishing group of generals Bonaparte gathered about him when Europe was one great battlefield, but he would have found it far more difficult to undertake the task of an American school teacher than to defeat the forces of Suwarrow, Wurmser and Wellington.

Nay, more. The North Carolina Ney got drunk. Napoleon's Ney was noted for his austere temperance. He never touched intoxicating drink. The claim that the tippling North Carolina Ney was the real Ney is too ridiculous for elaborate refutation. Still it is time the silly story should be shown up in such a shape as to silence it forever.

